# On the Nervous Disturbances of Pellagra and Ustilago Maides

**Published:** 1888-08

**Authors:** 


### ON THE NERVOUS DISTURBANCES OF PELLAGRA AND USTILAGO MAIDES.

Professor Tuizeck, of Marbery, visited Lombardy and Venice in order to study pellagra at its usual home, and he found that among ten thousand pellagra patients ten per cent. are mentally alienated, and ten per cent. succumb to the disease. He studied three hundred and fifty cases, and participated in eight autopsies.

During the first stage of the disease we meet great muscular debility, erythema on the back of the hand and mental debility. Thus the first attack may close, till in the following spring all symptoms reappear, but those of the central nervous system become worse. Even now the patient may recover. During the third year all manifestations reappear, and now the mind begins to suffer. The morbid manifestations of the first stages vary greatly according to the prevalence of spinal or cerebral symptoms. In the latter stages mental functions are always greatly disturbed, and *melancholie cum stupore* is the rule. The patient shows a peculiar look characteristic of the disease. In a few cases maniacal states were observed or manifestations reminding one of paralysis, or twitchings in various parts, as in epilepsy, but no genuine epileptic attacks. Among the spinal manifestations paraesthesia deserves mentioning, and such an unbearable burning, itching of the skin that it may lead to suicide, sensation of globus, cervical pains, sensation of constriction as from a cord. As disturbances of motility we meet general malaise, muscular spasms, in more severe cases twitching and cramps of the muscles. Patellar reflex was studied on three hundred cases, and found increased in about two-thirds of the cases, sometimes to such a degree as showing patellar clonus. In twenty-three cases the foot-clonus was intense, corresponding to the picture of spastic spinal paralysis. In seven cases the patellar reflex was absent. Among the vasomotory disturbances cutis anserina must be mentioned; among the secretory disturbances inhibition of the perspiratory function and of other secretions.

Of great importance is the exanthema. It is an erythema of the uncovered parts of the body, especially of the hands; though it is also found on the covered parts, and it may disappear after a few months. After every relapse residua remain, the skin becomes like parchment, dark, drawn, and rhagades from under the cutis. A further trophic disturbance we find on the tongue, which becomes fissured and devoid of epithelium. We deal here with a chronic intoxication, analogous to ergotism.

Our results from the autopsies are not quite ready. Whereas, of the Italian authors, Tonine found changes in the posterior columns, with co-affection of the lateral columns, Tuizeck found a combination of affections of the posterior columns, and of the posterior lateral columns. The gray substance, Clark's columns, are nearly always intact. The affection of the white matter is in all cases bilaterally symmetric.

Corpora amylacea in large quantities. Anatomical examination confirmed the analogy with ergotism, but in the latter the posterior columns are affected, in lathyresmus the lateral columns, whereas in pellagra we meet a combined affection of the posterior and posterio-lateral columns. Whether the disease has anything to do with micro-organisms, which course in the blood, and thus reach the nervous system, is more than doubtful. —*Centralbl. f. Nervenheilkunde*, 19*th*, 1887.

Let us try now to study the symptoms of Ustilago as given here by Professor Tuizeck, and compare them with those of Secale cornutum, and Lathyrus. In all three we find similarity, but certainly the acting poison differs greatly in each of them. We will finally act on Professor Allen's hint, and compare also Lathyrus with Calabar bean.

Ergot and Ustilago show a marked action upon the brain and spinal chord, but in Ergot we find at first stimulation, soon followed by paralysis, whereas, under Ustilago mental deterioration shows itself from the very start as mental hebetude, to be increased in the third attack to a state bordering on idiocy, while under Lathyrus we meet no mental symptoms recorded, nor does Physostygma give us any disturbance of the mind. The acting poison of these beans must differ therefore materially from those found in sweet rye or sweet corn.

The disturbances of motility found in pellagra consist in general malaise, muscular debility, and in severe cases muscular spasms and cramps. Corresponding to it we read in our provings "very languid for a number of days after the proving; number of faint spells, commencing in epigastrium, felt oppressed for want of fresh air; frequent rheumatic (far more neuralgic?) pains in the upper and lower extremities, tingling sensation in right *hand and arm.*"

Wood, in his *Materia Medica*, page 526, gives us the symptoms of ergotism: violent and painful tonic contractions, affecting especially the flexor, gradually increasing to tetanic paroxysms, with death from exhaustion, also gangrenous ergotism, beginning by tingling numbness and formication, an insupportable sense of fatigue, an earthy hue of the skin, coldness of surface.

In Lathyrus, on the contrary, we find only paralytic symptoms of the *lower extremities*, with tremulous, tottering gait, sensibility remaining intact, and during rains followed by emaciation of the affected limbs, while the upper extremities retain their natural appearance, shutting their eyes while standing or walking in no wise modified their attitudes and movements.

That other bean, Physostygma, acts in a similar manner as Lathyrus, for Bartholow in his *Materia Medica*, page 412, says of it: "The Calabar bean does not affect the centres of conscious impressions, and consciousness is preserved until the oxygenation of the blood is so far interfered with that carbonic acid narcosis supervenes. Giddiness, vertigo, and a sense of muscular weakness are produced by considerable doses. When a lethal dose is given complete paralysis ensues. The voluntary muscular system before complete resolution occurs is agitated by a succession of tremors—temporary tetanic contractions, followed by entire relaxation. Muscular contractility is not even accompanied by Physostygma and the sensibility of the sensory nerves is rather heightened, and Dr. W. P. Wesselhœft (Allen, vii, 496) says : "The chief action of the drug seems to be in the nerves and showing a very curious double action, exciting the nerves in the front of the body to involuntary motion, causing trembling and going up and down in wave-like motions, but the nerves in the back of the body it seems to paralyze and numb, and during this numbing process it causes a good deal of pain."

*Ustilago* gives us paraesthesia of the skin, unbearable itching or burning of the skin. *Secale*, formication, with a sense as if mice were creeping under the skin, crawling in the tips of fingers, hands, neck, and other parts, as if the parts had been aching, and sensation returning with returning warmth. The sensory nerves are unaffected in Lathyrus and Calabar beans—in fact, in relation to nervous symptoms are awfully deficient, and except to Ustilago we hardly even know whether the tendon reflexes are normal or abnormal.

Our two first drugs show most severe influence on the skin, Ustilago producing an itching, burning erythema (reminding one of its neural origin) of the uncovered parts of the body, though it may also appear on the covered parts, and leaving by and by the skin parchment-like—dark brown and with rhagades under the skin. *Secale*, dry and withered appearance of the skin and of a muddy, yellow hue, the parts gradually become numb, and lose all sensibility. This dry gangrene is often preceded by great burning, itching of the skin, and scratching only increases the unbearable heat. In relation to skin symptoms we find nothing mentioned under Lathyrus, as no proving was ever made, and under Physostygma we only read of petechial eruption (somewhat similiar to Secale) with a pricking sensation all over the affected parts.

The most interesting part to me was the revelation given by the autopsies, and they fully deserve our close attention. Tontini and Tuizeck found in the Ustilago pellagra a combined affection of the posterior and posterio-lateral columns, the gray substance and Clark's columns intact. In ergotismus the posterior, in lathyrismus the lateral columns are affected. Ustilago ought, therefore, to be a valuable remedy in spastic paralysis, in spinal paralysis of infants and adults, followed by atrophy, whereas Secale might be thought of in the first stages of locomotor ataxia, when the pains are so often wrongly diagnosed as rheumatic ones. Lathyrus ought certainly to be tried in those sudden cases of paralysis, coming as it were over night, and being entirely of spinal or peripheral origin. We know very little yet of ascending paralysis, and in relation to the homœopathic application of our remedies our *Materia Medica* needs overhauling, and this more so as the whole study of spinal affections dates back only a few decades. What a difference between the pathology of the first fifty years of this century and that of the present, and how much has been done in the latter part of it, and how much must be done yet to clear up the dark corners! If drug therapeusis is our branch, let us see to it that we progress *pari passu* with the other laborers in other branches, for only then we perform our part as faithful and progressive homœopathic physicians. S. L.

